# A single-centre retrospective study on the clinical characteristics of patients with hereditary angioedema and the therapeutic effect of lanadelumab

**DOI:** 10.1186/s13023-025-03988-7

**Published:** 2025-08-18

**Authors:** Yanhua Xu, Yinshi Guo

**Affiliations:** Department of Allergy, Renji Hospital, Shanghai Jiao Tong University School of Medicine, 200001 Shanghai, China

**Keywords:** Hereditary angioedema, Clinical characteristics, Lanadelumab, Efficacy, Safety

## Abstract

**Background:**

Hereditary angioedema (HAE) is a rare monogenic disease, and there are few reports on its clinical characteristics, particularly its drug efficacy, in China. The objective of this study was to gain insight into the clinical characteristics of HAE in Chinese patients, and the efficacy and safety of prophylactic treatment with lanadelumab.

**Results:**

The cohort included 22 patients with a median age of 35.0 years (IQR: 27.0–48.3 years). The male-to-female ratio was 1:1.75. The median age at onset was 15.5 years (IQR: 10.0–21.3 years), with a median diagnostic delay of 18.5 years. A significant positive correlation was found between patient age and the duration of diagnostic delay (*r* = 0.750; *p* = 0.000). In the cohort, 18 patients (81.8%) had Type I HAE, whereas 4 patients (18.2%) had Type II HAE. The average monthly frequency of attacks was 1.0 (IQR: 0.3, 1.3). Ten patients (45.5%) experienced onset following minor trauma/local bumps/pressure/heat exposure, which was the most common precipitating factor; 7 patients (31.8%) experienced spontaneous onset without apparent precipitating factors. A family history was reported for 16 patients (72.7%). Six patients (27.3%) had concomitant diseases involving various positive autoantibodies or confirmed autoimmune diseases. Eleven patients (50.0%) in this cohort were either currently receiving or had previously received lanadelumab treatment, with a median treatment duration of 7 months (IQR: 3–10 months). Nine patients reached the steady-state period of treatment (> 70 days). Eight patients experienced no oedema attack during treatment. There was a significant reduction in the frequency of attacks and a significant improvement in quality of life by Day 30 (D30) posttreatment, with a decrease of 91.5% in the average monthly frequency of attacks. The average monthly frequency of attacks decreased by 94.6% and 96.2% after 3 months of treatment and at the time of the last injection, respectively, with no life-threatening laryngeal oedema attacks. Only 5 patients (45.5%) experienced local adverse reactions during treatment, and no severe adverse reactions were reported.

**Conclusion:**

(1) The median age at onset, diagnostic delay, and precipitating factors in this cohort were consistent with previously reported data from domestic studies. However, the proportion of Type 2 patients was greater than that in prior domestic reports, and a trend towards earlier diagnosis in younger patients was observed; notably, this cohort identified a high proportion (27.3%) of patients with positive autoantibodies or confirmed autoimmune diseases for the first time in China. (2) After treatment with lanadelumab, patients experienced significant improvements in symptoms, quality of life, and anxiety/depression levels. Symptom control was achieved by D30 prior to the drug steady state-period and was maintained throughout the entire treatment period. No serious adverse reactions were observed during the treatment, indicating a high safety profile for the medication.

**Trial registration:**

Chinese Clinical Trial Registry, ChiCTR2500098307, 2025/03/05.

## Background

Hereditary angioedema (HAE) is a rare autosomal dominant disease that is clinically characterized by recurrent, nonpruritic, oedema of subcutaneous or submucosal tissues, often involving the extremities, face, gastrointestinal tract, and even the throat [1, 2]. The global prevalence of HAE is uncertain, with reported prevalence ranging from 1/100,000 to 1/50,000 in different regions [3]. On the basis of available data, the overall prevalence across Asian Pacific countries is estimated to be 0.02/100,000 people but has a large range (from 0.02 to 0.33/100,000 people) [4]. HAE can be classified into three types on the basis of the underlying pathogenesis: deficient C1INH (HAE-C1INH Type 1, HAE Type 1), dysfunctional C1INH (HAE-C1INH Type 2, HAE Type 2), and normal C1INH (HAE-nC1INH, HAE Type 3) [5]. HAE-C1INH-Type1 and HAE-C1INH-Type2 are associated with mutations in the *SERPING1* gene. In contrast, HAE-nC1INH is relatively rare, with the currently identified genes related to it including *FXII*,* ANGPTI*,* PLG*,* KNG1*,* HS3ST6*,* MYOF*,* CPN*,* and DAB2IP* [6]. Lanadelumab is currently one of the first-line options for long-term prophylaxis (LTP) and is the only first-line LTP drug approved in China. Jane reported the effects of this drug on 3 patients with HAE in Hong Kong [7], whereas Yao presented a multicentre study conducted on 6 patients in mainland China [8]. This study summarizes the clinical characteristics of 22 HAE patients who visited our department, and provides a preliminary analysis of the efficacy and safety of lanadelumab in 11 patients in our cohort.

## Subjects and methods

### Subjects

A total of 22 HAE patients (from 17 families) who visited our department from July 2022 to July 2024 were included in the study. Diagnosis and classification were performed according to the diagnostic criteria of the World Allergy Organization (WAO) Guidelines 2021 [9], which use clinical manifestations, laboratory tests, family history, and other relevant information. All Type 1 patients underwent at least complement C4 and C1INH concentration tests, which are routinely conducted in the laboratory of our hospital, as part of the diagnostic criteria. Since the C1INH function assessment must be performed at a specific inspection institution, Type 1 patients who are willing to complete C1INH functional testing, as well as patients suspected of being Type 2, underwent testing after providing informed consent. All tests were conducted with peripheral blood samples, not dry blood samples.

Among them, Type 1 patients exhibited both reduced C1INH concentration and C1INH function, with values falling below 50% of the normal range. Type 2 patients demonstrated a decrease in C1INH function to below 50% of the normal range, while their C1INH concentration could be normal or elevated.

### Methods

The clinical characteristics were summarized and analysed on the basis of the patient’s medical history, including age at onset, site of involvement, precipitating factors, frequency of attacks, family history, and concomitant diseases. The pre- and posttreatment efficacy in patients treated with subcutaneous injection of lanadelumab was also assessed, including the number of angioedema attacks in the 3 months before treatment, at Day 30, 3 months after treatment, and during the last injection period. Additionally, assessment was conducted via completion of the angioedema control test (AECT) score, the angioedema quality of life (AE-QoL) questionnaire, and the hospital anxiety and depression scale (HADS), and posttreatment adverse reactions were also recorded for treatment efficacy and safety analysis.

The AECT is a validated retrospective tool that contains 4 questions for assessing patients’ disease control over recurrent angioedema symptoms over the past 4 or 12 weeks [10]and is also applicable to HAE patients in China [11]. The assessment is based on cumulative scores, which differentiate between patients with inadequate disease control (minimum score of 0) and those with well-controlled disease (maximum score of 16) using a cut-off value of 10 [12].

The AE-QoL is a validated questionnaire used to assess quality of life in patients with recurrent angioedema [13, 14], which is also applicable to HAE patients in China [11]. It contains 17 questions that address a range of topics, including function, fatigue/mood, fear/shame, and nutrition. Each question is rated on a 5-point scale to evaluate severity, with the total score linearly transformed to a range of 0−100, where higher scores indicate a greater impact on quality of life.

The HADS is a validated questionnaire scoring tool used to assess symptom severity in anxiety states and depression states in patients or the general population [15]. Scores are interpreted as follows: 0−7 = normal; 8−10 = mild; 11−14 = moderate; and 15−21 = severe.

### Statistical processing

The data were summarized and analysed by SPSS 25.0 statistical software. Normally distributed variable data were expressed as x̄±s, and paired comparisons were performed using the *t* test. Nonnormally distributed variable data were expressed as M (IQR: P25−P75), and paired comparisons were performed using the Wilcoxon rank sum test. Statistical descriptions of attribute data were expressed as the number of cases (%). Correlations between two continuous variables were analysed using the Spearman correlation test. Statistical significance was defined as *P* < 0.05.

## Results

### Demographic data

**Fig. 1:**
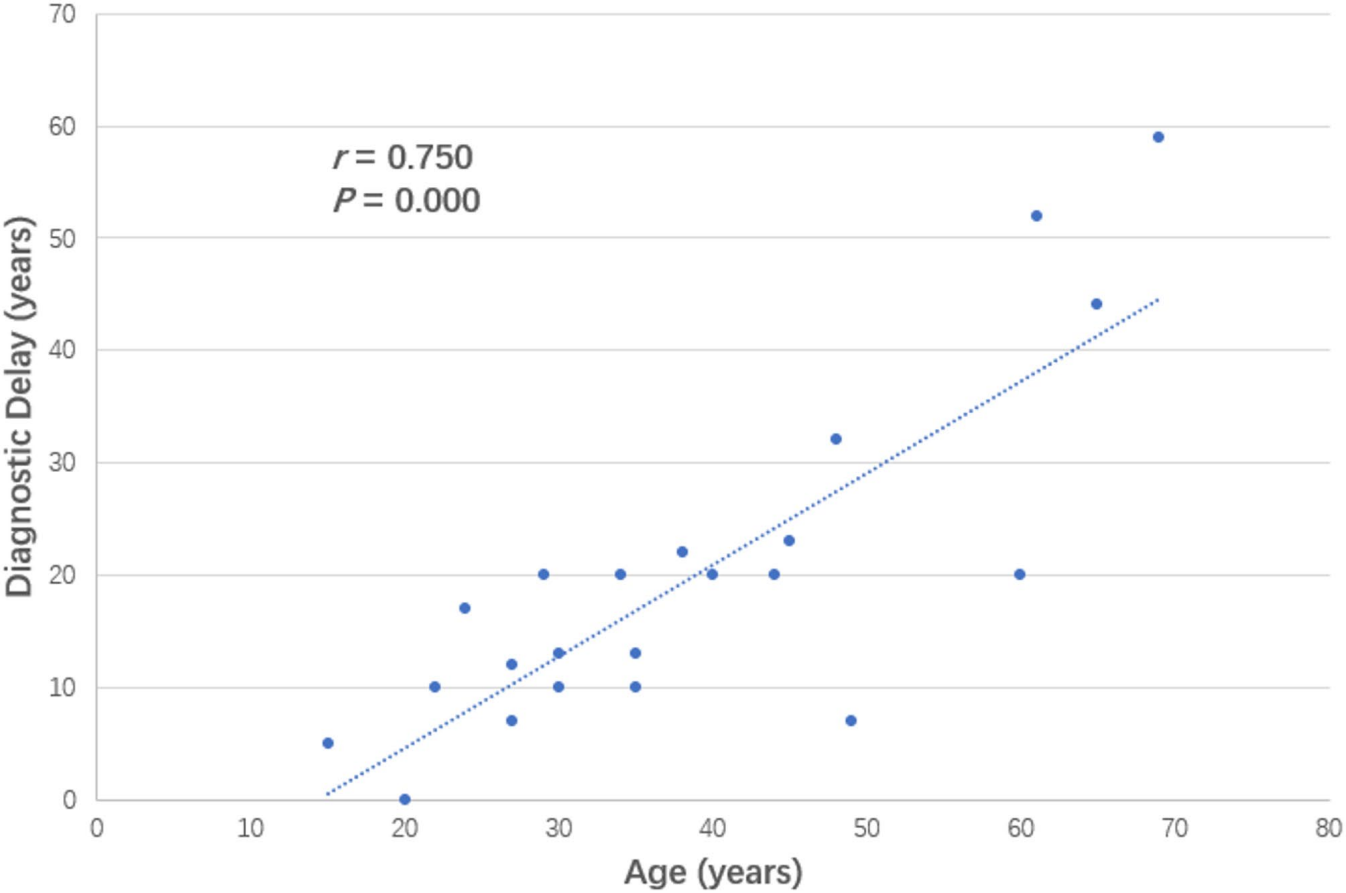
Correlation between patient age and duration of diagnostic delay

A total of 22 patients (from 17 families), including 8 males and 14 females, were included in the study. The median age was 35.0 years (IQR: 27.0−48.3 years). The male-to-female ratio was 1:1.75. The median age at onset was 15.5 years (IQR: 10.0−21.3 years), with an age at diagnosis of 34.5 years (IQR: 25.5−44.3 years)and a median diagnostic delay of 18.5 years. A significant positive correlation was found between patient age and the duration of diagnostic delay (*r* = 0.750, *P* = 0.000) (Fig. 1). Eighteen patients (81.8%) had Type 1 HAE, and 4 patients (18.2%) had Type 2 HAE. The average monthly frequency of attacks was 1.0 (IQR: 0.3, 1.3).

### Clinical characteristics (Table 1)

**Table 1:** Clinical characteristics of 22 HAE patients

	Sex/Age	Type	C4 (0.1–0.4 g/L)	C1INH concentration (0.21–0.39 g/L)	C1INH function ( ≧ 68%)	Age at onset/diagnosis	Site of involvement	Precipitating factor	Frequency of attacks	Family history	Concomitant diseases
Patient 1	Female/30	Type I	0.038	0.09	-	7/20	Face, extremities, abdomen, throat	Local infection, emotional stress	1 time per month	The father is a carrier but has not developed the disease	Allergic rhinoconjunctivitis, allergic dermatitis
Patient 2	Male/49	Type II	0.054	0.33	15.1%	27/34	Extremities, abdomen, throat	Upper respiratory tract infection, minor trauma	1 time in several months	None	Hypertension, hyperlipidaemia
Patient 3	Female/34	Type II	0.020	0.70	6.3%	14/34	Extremities, abdomen, throat	Local pressure	1−2 times per month	Two sons	None
Patient 4	Male/27	Type II	0.044	0.66	< 7.0%	20/27	Face, extremities, abdomen	Local pressure, diet	1 time in several months	Mother, aunt, uncle, sister	None
Patient 5	Female/61	Type I	0.110	0.07	0.0%	7/59	Face, extremities, trunk (armpits, buttocks, perineum), abdomen, throat	Sedentary, menstruation, local skin infection, oral operation, spontaneous	3 times per month	Mother, two sisters, son	Diabetes mellitus, ANA granular pattern at a titre of 1:80
Patient 6	Male/20	Type I	0.143	0.08	42.6%	20/20	Abdomen	Spontaneous	1 time	Son of the Patient 5	None
Patient 7	Female/24	Type I	0.030	0.07	-	7/24	Face, extremities, abdomen, throat	Spontaneous	1 time in several months	Grandfather, father	ANA homogeneous pattern at a titre of 1:80, granular pattern; weakly positive p-ANCA
Patient 8	Male/35	Type I	0.030	0.04	-	25/35	Extremities, scrotum, abdomen, throat	Fatigue	1 time per month	Father	Urticaria, allergic asthma, scoliosis
Patient 9	Male/30	Type I	0.050	0.05	0.0%	20/30	Extremities, buttocks, scrotum, abdomen	Fatigue	1−2 times per month	Two daughters	None
Patient 10	Female/40	Type I	0.020	0.03	2.6%	20/40	Face, extremities, throat	Local pressure, minor trauma, dental procedure	1−2 times per month	Grandmother	None
Patient 11	Female/38	Type I	0.032	0.03	0.0%	16/38	Face, extremities, abdomen	Fitness, dental procedure, emotional stress, fatigue, spontaneous		The mother is a carrier but has not developed the disease	Hypothyroidism, hyperprolactinemia
Patient 12	Male/60	Type I	0.060	0.04	-	40/60	Extremities, abdomen	Upper respiratory tract infection, diet	1 time every 2−3 months	Daughter, son, grandson	
Patient 13	Male/44	Type I	0.054	0.08	0.0%	24/44	Face, extremities, abdomen, throat	Dental procedure, diet	1 time per month	Grandfather, mother, aunt, uncle, cousin, sister, son	Hypertension, hyperlipidaemia, Hashimoto’s thyroiditis complicated by subclinical hypothyroidism (TPO-Ab 337.7 IU/ml, TG-Ab > 1000 IU/ml); ANA nuclear granular pattern at a titre of 1:80, Ro52:73 (+), anti-SSA-Ro60:72 (+), and weakly positive atypical ANCA
Patient 14	Male/15	Type I	0.097	0.07	2.4%	10/15	Abdomen	Diet	1 time in several months	Son of the Patient 13	Anti-PM-Scl antibody 34 (+)
Patient 15	Female/35	Type I	0.070	0.08	23.9%	22/35	Eyelids, extremities, abdomen	Menstruation	1 time per month	Grandmother, mother	Allergic dermatitis
Patient 16	Female/29	Type I	0.051	0.07	0.0%	9/29	Face, extremities, abdomen, throat	Emotional stress, spontaneous	1−2 times per month	Father	None
Patient 17	Female/65	Type I	0.040	0.04	0.0%	21/65	Face, extremities, abdomen, throat	Dental procedure, minor trauma, emotional stress	1 time per month	None	None
Patient 18	Female/69	Type I	0.010	0.02	0.0%	10/69	Face, extremities, throat	Bump	1 time per month	None	None
Patient 19	Female/48	Type I	0.017	0.09	1.2%	13/45	Face, abdomen	Bump	1 time in several months	Grandmother, mother, aunt, uncle, cousin, daughter	Systemic lupus erythematosus (SLE)
Patient 20	Female/22	Type I	0.044	0.06	0.0%	10/20	Face, extremities, buttocks, throat	Diet, local heat exposure	1 time per month	Daughter of the Patient 19	ANA dense fine speckled pattern at a titre of 1:80; Golgi pattern
Patient 21	Female/45	Type I	0.022	0.04	-	15/38	Face, extremities, abdomen, throat	Fatigue	1 time in several months	Cousin of Patient 19	None
Patient 22	Female/27	Type II	0.020	0.41	0.4%	14/26	Face, extremities, abdomen, throat	Emotional stress, fatigue, diet, contraceptives, spontaneous	1 time in 1−2 months	None	None

### Clinical symptoms

All patients experienced recurrent angioedema attacks at different sites. Twenty patients (90.9%) had attacks involving the extremities or face; 6 patients (27.3%) had attacks involving the perineum/buttocks/scrotum; and 19 patients (86.4%) experienced abdominal pain or discomfort, of whom Patient 6 had only one abdominal pain attack. Given that Patient 6’s mother (Patient 5) was confirmed, Patient 6 was rapidly diagnosed after onset, and no swelling was found at other sites in the patient’s past medical history; Patient 14, a direct descendant of Patient 13, was identified through familial screening and reported occasional mild abdominal discomfort; and 14 patients (63.6%) had laryngeal edema, of which 8 patients had ≥ 3 attacks.

#### Frequency of attacks

Twelve patients (54.5%) had ≥ 1 attack per month, 9 patients (40.9%) had an attack every 2−6 months, and 1 patient had only one attack to date. The overall average monthly frequency of attacks was 1.0 (IQR: 0.3, 1.3), with patients receiving lanadelumab having a mean of 1.3 (IQR: 0.7, 1.7) attacks per month and untreated patients having a mean of 0.6 (0.3, 1.0) attacks per month.

#### Precipitating factors

Ten patients (45.5%) experienced onset following minor trauma/local bumps/local pressure/heat exposure, which was the most common precipitating factor; 7 patients (31.8%) experienced spontaneous onset without apparent precipitating factors; 6 patients (27.3%) experienced onset that was diet-related (tea beverages, leftover food, alcohol, cold and raw foods); 5 patients (22.7%) experienced emotional stress, dental procedures, or fatigue as precipitating factors; 4 patients (18.2%) experienced attacks following upper respiratory tract infection/local skin infection; 2 patients (14.3% of females) experienced a correlation with their menstrual cycle; and 1 patient (4.5%) developed symptoms after fitness sessions (Patient 11 presented a history of severe abdominal pain following lower limb strength training during fitness sessions, necessitating hospitalization) or after administration of oral contraceptives (Patient 22 experienced limb swelling after taking oral contraceptives, possibly related to changes in oestrogen and progesterone levels).

#### Family history

All patients underwent family screening. Sixteen patients (72.7%) had a clear family history. For 2 patients (9.1%), family screening revealed that either the father or the mother was a carrier but had no clinical symptoms to date. Four patients (18.2%) had no family history.

#### Concomitant diseases

Two patients had concurrent hypertension/hyperlipidaemia (one of whom was treated with oral telmisartan and amlodipine to control blood pressure, and the medication was not related to his onset); one patient had diabetes mellitus (unmedicated, managed mainly through diet); one patient had simple hypothyroidism and hyperprolactinemia (treated with regular oral euthyrox and parlodel, respectively); two patients had allergic diseases (one patient had allergic rhinoconjunctivitis and allergic dermatitis and was treated symptomatically as needed; one patient had asthma, and was treated with salmeterol xinafoate and fluticasone propionate inhalation); one patient had scoliosis and had an acute urticaria attack, which resolved after short-term oral hormone therapy; one patient was definitively diagnosed with systemic lupus erythematosus and was currently treated with regular oral prednisone (5 mg) and hydroxychloroquine; and one patient was definitively diagnosed with Hashimoto’s thyroiditis complicated by subclinical hypothyroidism (TPO-Ab 337.7 IU/ml, TG-Ab > 1000 IU/ml, currently unmedicated). This patient also had an antinuclear antibody (ANA) nuclear granular pattern at a titre of 1:80, Ro52:73 (+), anti-SSA-Ro60:72 (+), and weakly positive atypical anti-neutrophil cytoplasmic antibodies(ANCAs). Despite these findings, no additional autoimmune diseases were diagnosed. Additionally, 4 patients presented various positive autoantibodies with insufficient evidence for a definitive autoimmune disease diagnosis upon consultation with rheumatology and immunology specialists. These included 1 patient with an ANA granular pattern at a titre of 1:80; 1 patient with an ANA homogeneous pattern at a titre of 1:80 and a granular pattern (+) along with weakly positive p-ANCA; 1 patient with positive anti-PM-Scl antibodies 34 (+); and 1 patient with an ANA dense fine speckled pattern at a titre of 1:80 and a positive Golgi pattern (+).

### Drug treatment

Compared with untreated patients, patients receiving treatment with lanadelumab presented differences in the frequency of attacks, AECT, and AE-QoL scores. The treatment group presented higher scores for anxiety and depression than did the untreated group; however, these differences were not statistically significant (Table 2).

**Table 2:** Comparison between patients treated with lanadelumab and untreated patients

	Monthly frequency of attacks(x̄±s)	AECT(x̄±s)	AE-QoL(x̄±s)	scores of anxiety(x̄±s)	scores of depression(x̄±s)
LTP (*n* = 11)	1.3(0.7, 1.7)	5.5 ± 3.8	56.5 ± 18.3	7.3 ± 4.7	5.5 ± 4.9
Non-LTP (*n* = 11)	0.6(0.3, 1.0)	11.0 ± 3.0	35.4 ± 11.0	4.6 ± 2.8	4.0 ± 2.0
*P* value	*P* = 0.037	*P* = 0.005	*P* = 0.015	*P =* 0.189	*P =* 0.442

Currently, 11 patients had not received LTP, including 8 patients (72.7%) who received icatibant on-demand treatment (ODT). Three patients were not prescribed icatibant for economic reasons. Eleven patients had previously received or were currently receiving lanadelumab, 9 of whom were treated for more than 3 months, with a median treatment duration of 7 months (IQR: 3–10 months). Among these 11 patients, 3 had previously received danazol treatment but discontinued it because of insufficient efficacy or safety concerns.

Among the 9 patients who had been regularly treated with lanadelumab for ≥ 3 months, the dosing regimens included 300 mg every 4 weeks (q4w) in 2 patients; 300 mg every 2 weeks (q2w) for 3 months followed by 300 mg q4w maintenance in 2 patients; and 300 mg q2w for 6 months followed by 300 mg q4w maintenance in the remaining 5 patients (Table 3).

**Table 3:** Summary of patients treated with lanadelumab

	lanadelumab treatment regimen	Total number of attacks in 3 months before treatment	Number of attacks 30−20days before treatment	Number of attacks 20−10days before treatment	Number of attacks 10−0days before treatment	Number of attacks 0−10days after treatment	Number of attacks 10−20days after treatment	Number of attacks 20−30days after treatment	Number of attacks 0−30days after treatment	Number of attacks 3 months after treatment	Number of attacks 3month - the last injection	Adverse reactions
Patient 1	300 mg q2w * 4 months and then discontinue	6	0	0	0	0	0	0	0	0	0	/
Patient 2	300 mg q2w * 3 months followed by 300 mg q4w * 14 months	1	0	0	0	0	0	0	0	0	0	Non-injection site pain
Patient 3	300 mg q4w * 9 months	4	0	0	0	0	0	0	0	0	0	/
Patient 4	300 mg q2w * 6 months followed by 300 mg q4w * 3 months	1	0	0	0	0	0	0	0	0	0	/
Patient 5	300 mg q2w * 6 months followed by 300 mg q4w * 7 months	10	0	0	0	0	0	0	0	0	2	Injection site pain, erythema, weight gain
Patient 9	300 mg q4w * 5 months	4	0	0	0	0	0	0	0	0	0	/
Patient 10	300 mg q2w * 6 months followed by 300 mg q4w * 2 months	4	1	1	1	1	1	1	1	0	1	/
Patient 11	300 mg q2w * 6 months followed by 300 mg q4w * 1 months	3	0	0	0	0	0	0	0	1	1	Injection site soreness, mild drowsiness
Patient 13	300 mg q2w * 3 months	5	0	0	0	0	0	0	0	0	0	Injection site pain
Patient 17	300 mg q2w * 1 months	3	0	0	0	0	0	0	0	0	Treatment less than 70 d	Injection site pain
Patient 22	300 mg q2w * 2 months and then discontinue	2	0	0	0	0	0	0	0	0	Treatment less than 70 d	/

### Efficacy of lanadelumab treatment

### Frequency of attacks

**Fig. 2:**
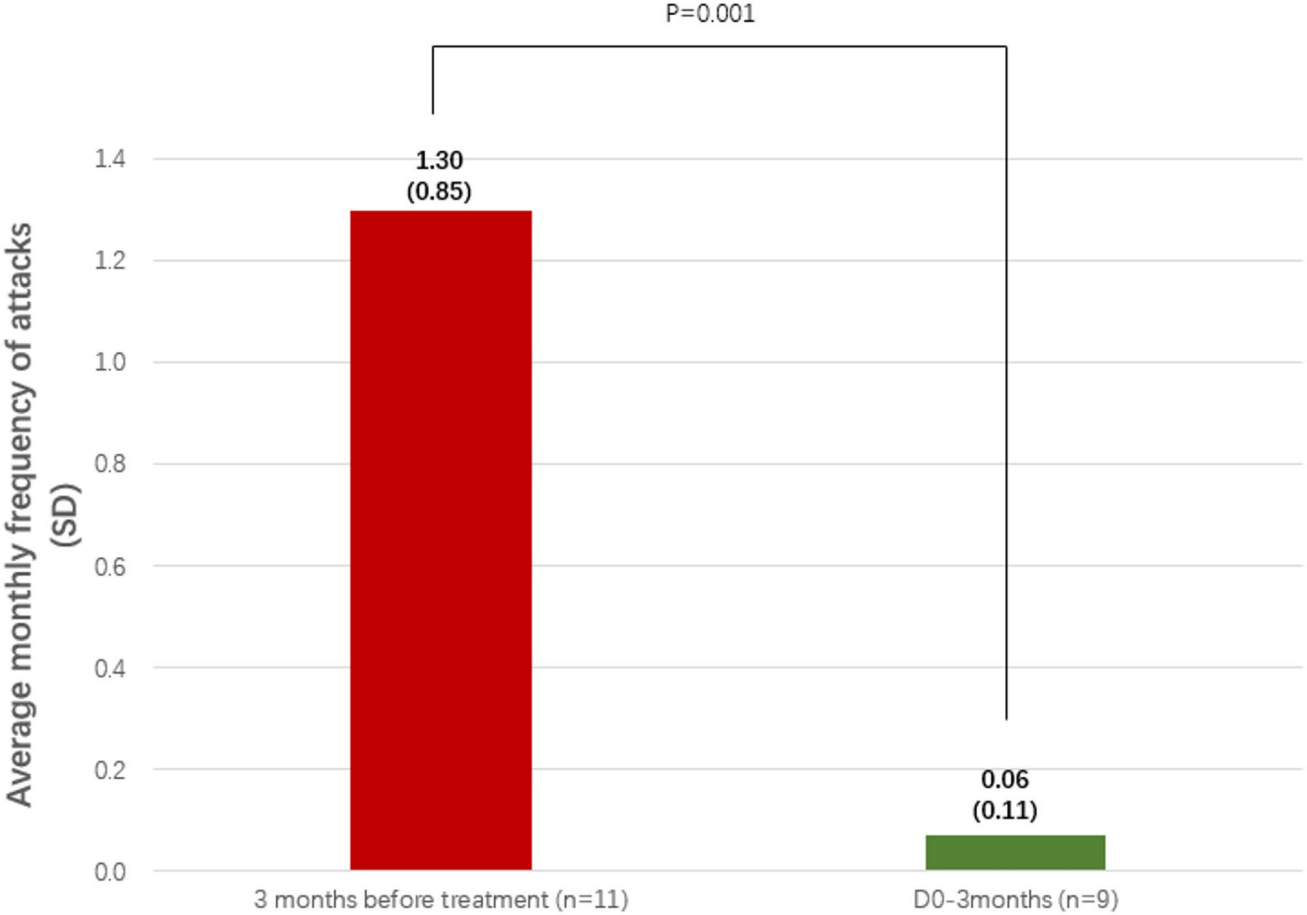
Comparison of average monthly frequency of attacks before and after treatment with lanadelumab

In the 3 months prior to regular treatment, the total number of angioedema attacks was 43 (including 1 abdominal pain attack and 2 laryngeal edema attacks, with the remaining attacks being swelling of the extremities, face, or trunk), and icatibant was used for 7 attacks. During the 30-day treatment period with lanadelumab, there was only 1 attack, resulting in a 91.5% reduction in the average monthly frequency of attacks. During the first 3 months of treatment, 9 patients experienced a total of 2 attacks, all of which experienced mild lower limb swelling that resolved spontaneously without medication, resulting in a 94.6% reduction in the average monthly frequency of attacks. During the treatment period after 3 months, 3 patients experienced a total of 4 attacks, resulting in an overall 93.8% reduction in the average monthly frequency of attacks; 2 of these patients each experienced a single attack of self-limiting swelling in the hands/feet, while 1 patient experienced a single attack of mild hand swelling and a single attack of moderate abdominal pain requiring icatibant intervention; there were no attacks of laryngeal oedema. Among the 11 patients who started treatment with lanadelumab, 8 patients (72.7%) experienced no oedema attacks during treatment, although the current treatment period varied. (Fig. 2)

Two patients discontinued lanadelumab treatment for different reasons. Patient 1 discontinued lanadelumab due to pregnancy after 4 months of treatment; and experienced recurrent swelling symptoms 5 months later, with 3–5 attacks per month, mainly involving the extremities, and 1 abdominal attack, that was relieved after the use of recombinant C1INH. Patient 22 discontinued the drug due to inconvenience in seeking medical care after 2 months of treatment and experienced mild swelling of the hands 2 months after discontinuation. (Table 3)

#### AECT score

**Fig. 3:**
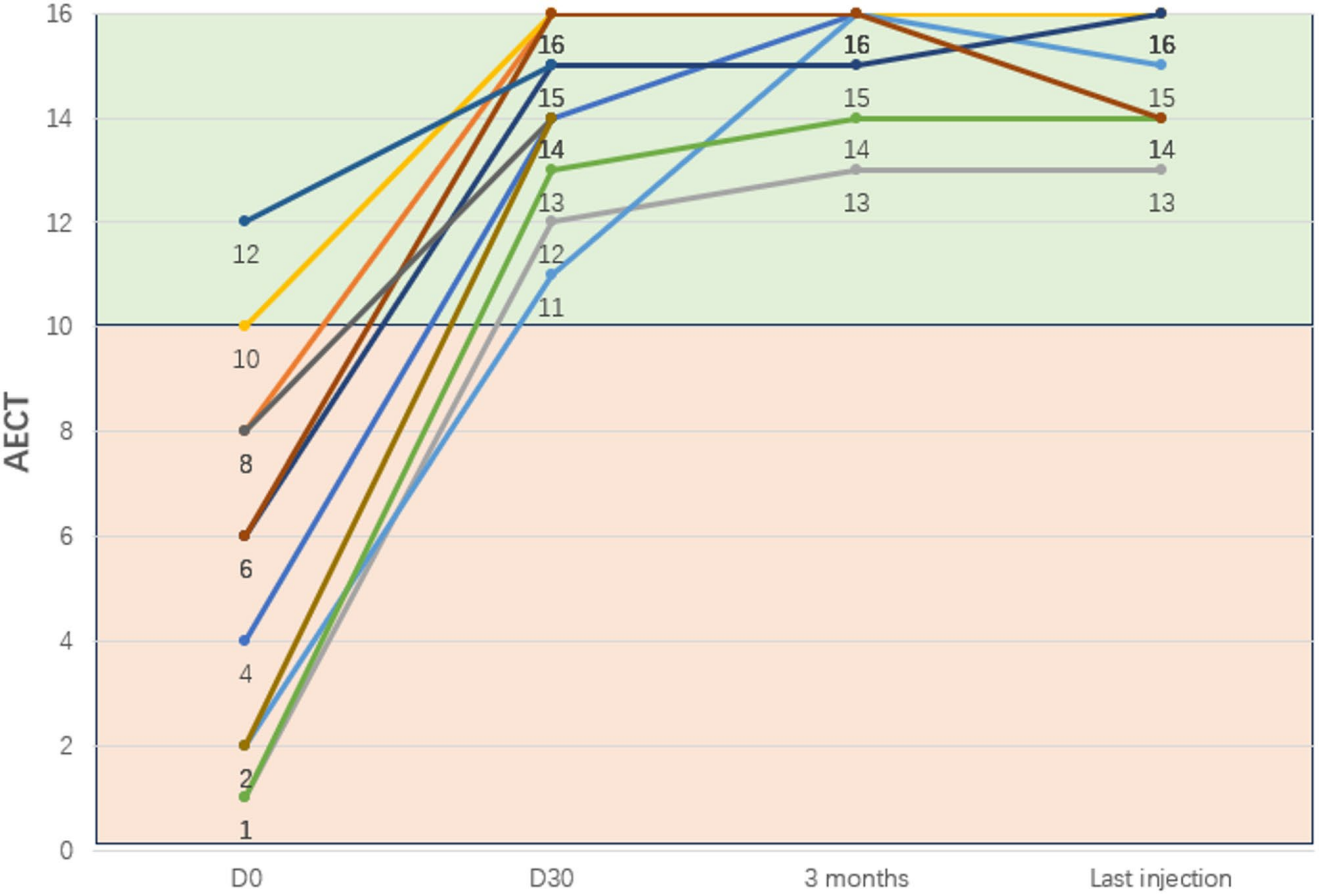
AECT score before treatment, and at Day30, 3 months, and the last injection after treatment with lanadelumab

The pretreatment AECT score for patients receiving lanadelumab was 5.5 ± 3.8 and increased to 14.2 ± 1.7 on Day30, and all patients achieved “good control” (AECT > 10 points); The score after 3 months of treatment was 15.3 ± 1.2 and remained stable (15.0 ± 1.2) until the last injection (Fig. 3).

#### AE-QoL score

**Fig. 4:**
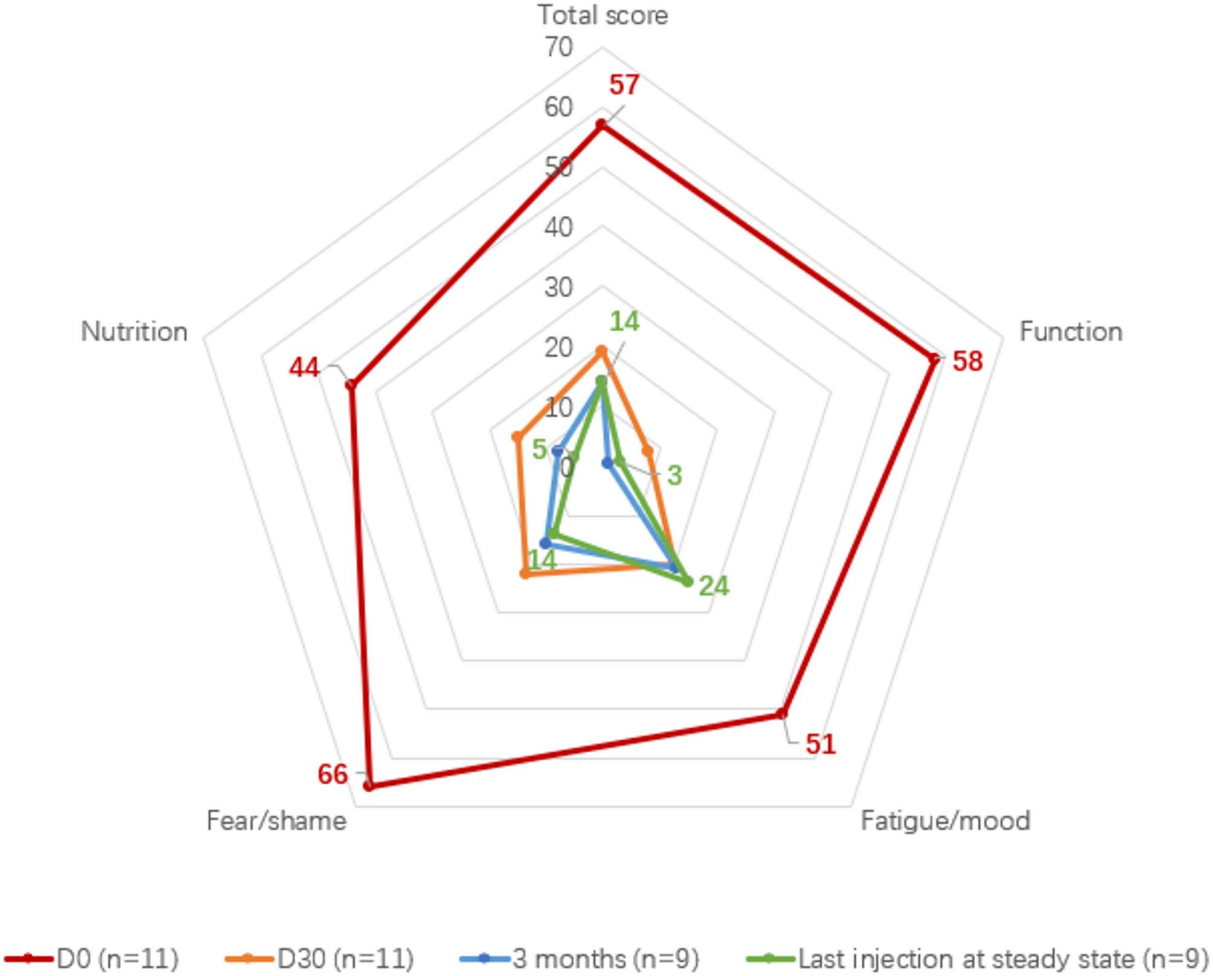
Comparison of quality of life scores before and after treatment with lanadelumab

The overall AE-QoL score and all domain scores (including function, fatigue/mood, fear/shame, and nutrition) decreased significantly in patients receiving lanadelumab. Significant differences were observed from baseline on Day30 (*P* ≤ 0.05), with the greatest improvement in the function domain. The fear/shame domain had the greatest impact on patients at baseline (Table 4; Fig. 4).

**Table 4:** Comparison of quality of life scores before and after treatment with lanadelumab

AE-QoL	D0	D30	3 months after LPT	Last injection	*P*
Total score	*n* = 11	*n* = 11	*n* = 9	*n* = 9	
$\bar x$ *±s*	56.5 ± 18.3	19.2 ± 15.0	14.3 ± 12.4	13.8 ± 14.8	D0 to D30: *P* = 0.000
M (Range)	51 (32–100)	19 (0–46)	15 (0–32)	9.5 (0–35)	
Function	*n* = 11	*n* = 11	*n* = 9	*n* = 9	
$\bar x$ *±s*	55.9 ± 21.8	7.9 ± 15.9	1.0 ± 1.9	2.6 ± 4.6	D0 to D30: *P* = 0.000
M (Range)	50 (25–100)	0 (0–50)	0 (0–4)	0 (0–13)	
Fatigue/mood	*n* = 11	*n* = 11	*n* = 9	*n* = 9	
$\bar x$ *±s*	50.9 ± 25.6	19.5 ± 18.0	20.6 ± 17.8	23.8 ± 23.1	D0 to D30: *P* = 0.005
M (Range)	50 (20–100)	25 (0–55)	25 (0–50)	25 (0–60)	
Fear/shame	*n* = 11	*n* = 11	*n* = 9	*n* = 9	
$\bar x$ *±s*	65.5 ± 19.0	21.7 ± 18.5	16.3 ± 16.1	14.0 ± 18.1	D0 to D30: *P* = 0.000
M (Range)	71 (25–100)	17 (0–58)	13 (0–46)	4 (0–46)	
Food	*n* = 11	*n* = 11	*n* = 9	*n* = 9	
$\bar x$ *±s*	44.3 ± 29.2	14.8 ± 16.6	7.8 ± 11.5	4.7 ± 9.3	D0 to D30: *P* = 0.002
M (Range)	50 (0-100)	12.5 (0–50)	0 (0–25)	0 (0–25)	

#### HADS score

**Fig. 5:**
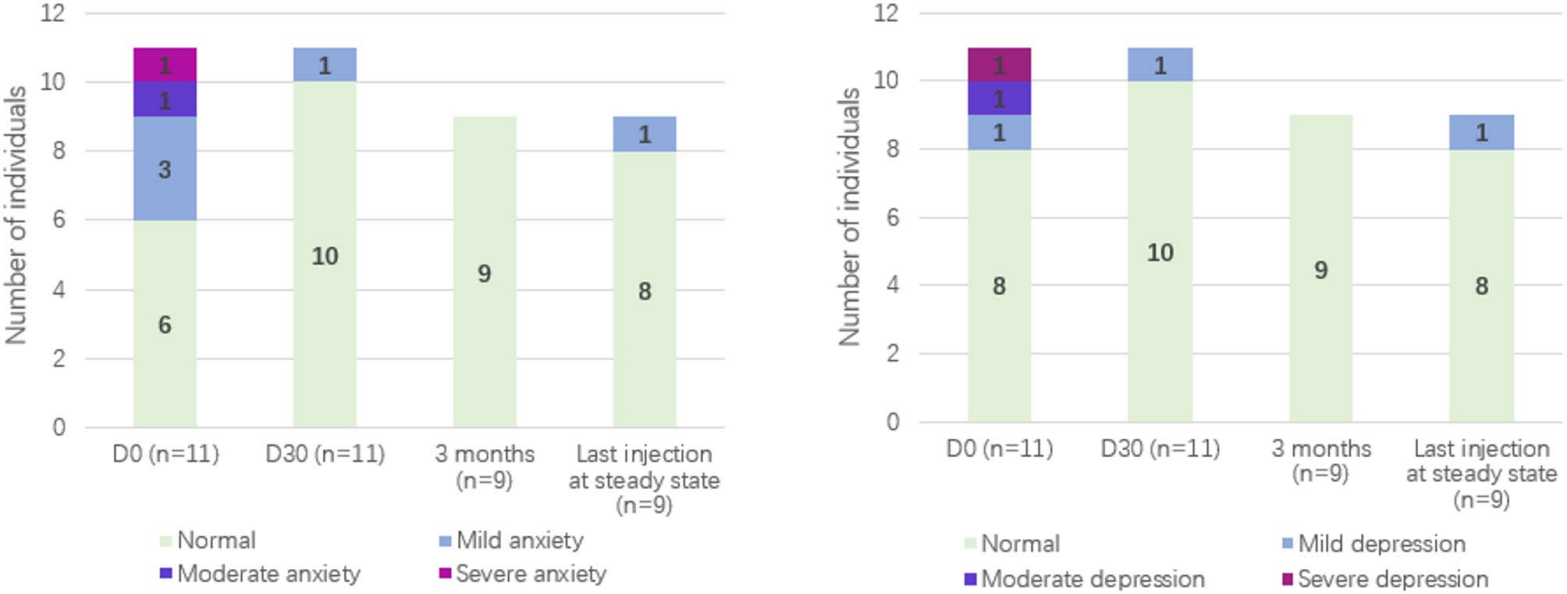
Comparison of anxiety/depression status during treatment with lanadelumab

Prior to treatment, 45.5% of the patients had anxiety and 27.3% had depression tendencies. Compared with those in the pretreatment period, the psychological states of patients in the treatment period improved, with the proportion of anxiety/depressive states decreasing to 9%. The influence on mental state before treatment is derived mainly from the disease itself. For example, Patient 5 in this cohort changed her career plan due to oedema attack at a young age, which was the patient with the highest frequency of attack and severity in this cohort; however, after treatment, even when the disease was basically stable, the mental state still fluctuated. Patient 5 expressed concerns regarding the potential impact of the disease on her offspring; Patient 4 expressed safety concerns about the long-term use of drugs; and Patient 11 expressed concerns regarding pregnancy preparation (Fig. 5).

### Safety of lanadelumab

None of the patients treated with lanadelumab experienced serious adverse reactions or discontinued treatment because of adverse reactions. Four patients (36.4%) experienced injection site pain, of whom 1 patient (9.1%) experienced local erythema that resolved within 30 min, as well as a weight gain of 5 kg during the first 3 months of treatment, which did not rise further; one patient (9.1%) experienced mild drowsiness following the first 2 injections, which did not recur after the third injection; and one patient (9.1%) experienced a single attack characterized by noninjection site pain (behind the ear), which resolved spontaneously and did not recur further. No patient abandoned treatment because of the above adverse reactions (Table 3).

## Discussion

This retrospective study was conducted to summarize and analyse the clinical characteristics and treatment status of 22 HAE patients (from 17 families) who visited our department from July 2022 to July 2024.

In this cohort of 22 patients, 81.8% had Type 1 HAE, and 18.2% had Type 2 HAE. The proportion of Type 2 patients was greater than that previously reported in China [16], but was similar to that reported internationally. This may be attributed to the further development of current clinical testing methods, especially C1INH functional testing. At present, the majority of hospitals employ complement C4 levels as the primary screening index for highly suspected HAE patients. Furthermore, some hospitals perform routine C1INH concentration testing, which is of significant diagnostic value for Type I HAE. In recent years, the further development of C1INH function testing has further increased the rate of Type 2 HAE detection. In regions or countries lacking resources for routine testing of C1INH function, patients exhibiting low C4 levels alongside elevated C1INH levels may be considered for Type 2 HAE screening, and should be prioritized and referred to a facility for confirmatory testing allowing for more targeted screening [17].

HAE follows an autosomal dominant inheritance pattern with incomplete penetrance and variable inheritance and phenotype [18, 19]. Family screening of 2 patients in this cohort identified carriers (the father in one case and the mother in the other) with the same gene mutation or abnormalities in complement C4 and C1INH levels and function. Despite these findings, neither carrier exhibited clinical symptoms. This suggests that even within the same family, differences in clinical symptoms can occur. Patient 6 is a direct relative of Patient 5, who never experienced symptoms such as limb or facial swelling and therefore declined family screening after Patient 5 was diagnosed. However, the first attack in Patient 6 presented as severe abdominal pain with ascites, leading to a rapid diagnosis. These results indicate that clinical symptoms are not a reliable sole predictor of disease, particularly in the case of asymptomatic offspring. Instead, predictions of future disease onset should be based on biological markers or genetic testing results, and emergency plans are crucial.

There was a trend towards early diagnosis and a shorter delay in diagnosis in younger patients in this cohort (Fig. 1), similar to the literature [20, 21]. Patient 14 had atypical clinical symptoms, but his prompt diagnosis benefited from family screening of the proband, in contrast to patient 6. This phenomenon may reflect an increase in patient awareness and in the ability of medical services, such as test-based diagnostics. The timely and accurate diagnosis of HAE may directly affect whether patients can receive the correct treatment and the clinical prognosis. Therefore, an in-depth understanding of the disease by both doctors and patients and an emphasis on family screening are extremely important for the clinical management of HAE.

In our cohort of patients, we observed for the first time that 6 patients (27.3%) had concomitant diseases involving various positive autoantibodies. One of these patients has been definitively diagnosed with SLE and is currently stable under treatment and follow-up. Another patient was diagnosed with Hashimoto’s thyroiditis. Multiple studies [22–26] suggest a significantly increased risk of autoimmune disease in HAE patients, indicating that screening should be enhanced for patients with clearly diagnosed autoimmune disorders if patients present with unexplained oedema to increase rates of detection. The limited sample size in this cohort calls for further investigations in larger patient populations with longer follow-up periods to clarify the clinical outcomes of patients with positive autoantibodies.

HAE is currently incurable, but can be managed through ODT, short-term prophylaxis (STP), and LTP to alleviate symptoms or reduce the frequency of attacks [27]. The primary goal of HAE treatment is to achieve complete disease control and restore the patient’s quality of life [9], which can only be accomplished through LTP. Before the domestic approval of lanadelumab in China, androgens were commonly used as prophylactic therapy for HAE patients to decrease the frequency and severity of HAE episodes [27]. However, concerns regarding their efficacy and long-term side effects have raised apprehensions among both physicians and patients. For this reason, some patients in this cohort switched to lanadelumab treatment.

At present, lanadelumab is the only first-line LTP drug available in China. It is a fully humanized monoclonal antibody that specifically inhibits active plasma kallikrein to prevent excessive bradykinin production [28]. The drug has a half-life of approximately 2 weeks [28, 29]; it is expected to reach a steady-state within 70 days (approximately 5 times the half-life) [30].

In this study, 11 patients underwent varying courses of lanadelumab treatment. Among them, 9 patients reached the steady state-period of treatment (> 70 days). Eight of these patients (72.7%) experienced no oedema attack during treatment. The average monthly frequency of attacks decreased by 91.5%, 94.6%, and 93.8% on Day30, 3 months after treatment and at the last injection, respectively. The reduction in the frequency of attacks was more pronounced on Day 30 than in the HELP study (70% reduction in the monthly frequency of attacks with 300 mg q2wks on Day30 of treatment in the HELP study) [30], which may be related to the variable frequency of attacks in patients before treatment. Patients in each group of the HELP study had an average of 3.2 to 4.0 monthly attacks, while patients in this cohort had an average of 1.3 monthly attacks, indicating that the overall frequency of attacks was lower than that in the HELP study. This may have facilitated the achievement of good disease control, leading to deviations in the results.

The initiation of LTP for patients with HAE varies across different countries. According to the guidelines [5, 31], the frequency of attacks is only one of many factors that should be considered, and the special needs of an individual patient should be considered. According to the consensus of domestic experts in China [32], if a patient’s quality of life is significantly affected—especially in cases involving multiple important organ systems, such as a history of abdominal pain or laryngeal oedema requiring emergency intervention—the initiation of LTP treatment may be suggested.

However, some patients are still advised to initiate LTP treatment on the basis of their symptoms but cannot do so owing to economic constraints. China’s expansive territory presents significant economic disparities, leading to substantial variations in health care infrastructure and insurance coverage across provinces and cities. Access to medications remains limited in numerous regions, particularly in second- and third-tier cities as well as remote areas. Once diagnosed locally, patients may struggle to adhere to regular medication regimens because of an inadequate drug supply in their place of residence. Furthermore, interprovincial prescription of medications may impose substantial economic burdens due to discrepancies in medical insurance policies. This situation contributes to the challenges faced by several patients in this cohort in initiating lanadelumab treatment or achieving the standby status for icatibant, highlighting the objective barriers that prevent adequate treatment of many rare diseases.

Although the overall frequency of attacks is lower than that in the HELP study and the treatment protocol was not the same, we still observed a reduction in the frequency of attacks that was similar to that in the HELP study. Despite Day 70 being the drug steady state-period, the treated patients showed a reduction in the frequency of attacks by Day30 of treatment (after 1–2 injections), which continued until the last injection. Two patients who chose 300 mg q4wks as the initial regimen for economic reasons also achieved good disease control with zero attacks posttreatment.

Patient 11 in this cohort discontinued lanadelumab due to pregnancy. At present,, there is no commercial pdC1INH or rcC1INH available for use during pregnancy in China. There are no large-scale data on the use of lanadelumab during pregnancy, and danazol is contraindicated during pregnancy. Therefore, disease management during pregnancy preparation, pregnancy and delivery appears to be more difficult. In Hong Kong, the Hospital Authority approved Berinert (pdC1INH) as a ‘special drug’ in 2023 for use by citizens suffering from HAE-C1INH, offering an alternative to LTP for more patients [33]. Over the past decade, rare diseases have gained increasing attention and priority from the central government and have been incorporated into strategic nationwide health plans. Both lanadelumab and icatibant have been included on the National Reinfusion Drug List, significantly improving the availability and accessibility of HAE-specific medications. However, owing to China’s large population, increasing the rate of HAE detection and ensuring adequate treatment relies on strengthening medical practitioners’ understanding of HAE at all levels and narrowing disparities in access to diagnostics and medications. Owing to its unique advantages, the The Greater Bay Area (GBA) in China has made significant efforts to promote HAE management and bridge the gap between East and West by establishing a ‘4A’ framework of ‘Awareness, Access, Advocacy, and Alliance’, which serves as a model for providing optimal patient care and advancing HAE management.

Lanadelumab has been marketed in China for a short time. At present, there are still relatively limited data on the treatment of HAE patients with this drug in China, especially data on the efficacy of long-term treatment. The median duration of treatment in our cohort was 7 months, which is insufficient to make definitive judgment regarding the efficacy and safety of long-term treatment. A German study provided follow-up data on 32 patients treated with lanadelumab for up to 4 years [34], which demonstrated the efficacy and safety of long-term treatment with lanadelumab. Notably, 22 of these patients reported 147 medical procedures that could p increase the risk of oedema attacks since LTP treatment, including tooth extraction, dental implants, and gastroscopy. While all patients were prescribed adequate C1INH as an STP prior to these medical procedures, only two patients actually used it. Among the 147 exposed patients, 146 had no edema attacks in the absence of STP treatment. The researchers concluded that the necessity of STP as a strong recommendation requires further clinical evidence when regular LTP is effective. Patient 5 (laryngeal oedema induced by tooth extraction at a young age) and Patient 11 in this cohort both underwent tooth extraction/filling procedures without STP treatment during treatment with lanadelumab and did not develop edema. Considering the limited availability of STP options in China, long-term prophylaxis for patients with LTP may be able to address the need for STP in patients requiring LTP. However, further evidence-based medical support is still needed.

This study has several limitations. First, as a retrospective study, recall bias may exist during data collection. Moreover, the lack of gene mutation analysis is a limitation of our study. In addition, the relatively small sample size limits the generalizability of our findings, such as the absence of HAE-nC1INH patients in this cohort, and the exploration of HAE comorbidities with autoimmune diseases. Therefore, prospective studies with larger patient populations and longer-term follow-up as well as the exploration of treatment reduction strategies in the maintenance phase are the direction of our future efforts.

## Conclusions

The 22 HAE patients in our cohort may be predisposed to a high comorbidity of autoimmune diseases. Young HAE patients tended to be diagnosed earlier.

Lanadelumab is currently the only first-line LTP drug available in mainland China and has not been on the market for long. Eleven patients in our cohort received lanadelumab. It has shown superior efficacy and safety, but longer term efficacy monitoring remains to be performed.

## Data Availability

Not applicable.

## Abbreviations

AECT: Angioedema control test
AE-QoL: Angioedema quality of life
ANA: Antinuclear antibodies
ANCA: Anti-neutrophil cytoplasmic antibodies
C1INH: C1 INHibitor
HADS: Hospital anxiety and depression scale
HAE: Hereditary angioedema
LTP: Long-term prophylaxis
ODT: On-demand treatment
pdC1INH: Plasma-derived C1INH
PM-Scl antibody: Human polymyositis-sclerosis antibody
rcC1INH: Recombinant C1INH
STP: Short-term prophylaxis
TG-Ab: Thyroglobulin antibody
TPO-Ab: Anti-thyroid peroxidase antibody
WAO: World Allergy Organization
